# Supplementary material for: Oncolytic Virus Therapy Alters the Secretome of Targeted Glioblastoma Cells
Source: Cancers (Basel). 2021 Mar 14;13(6):1287. doi: 10.3390/cancers13061287 (PMC7999647; doi:10.3390/cancers13061287)
Supplement: Supplementary file 1 [file cancers-13-01287-s001.pdf]

cancers

MDPI

Oncolytic Virus Therapy Alters the Secretome of Targeted Glioblastoma Cells

Jakub Godlewski, Mohamed Farhath, Franz L. Ricklefs, Carmela Passaro, Klaudia Kiel, Hiroshi Nakashima, E. Antonio Chiocca and Agnieszka Bronisz

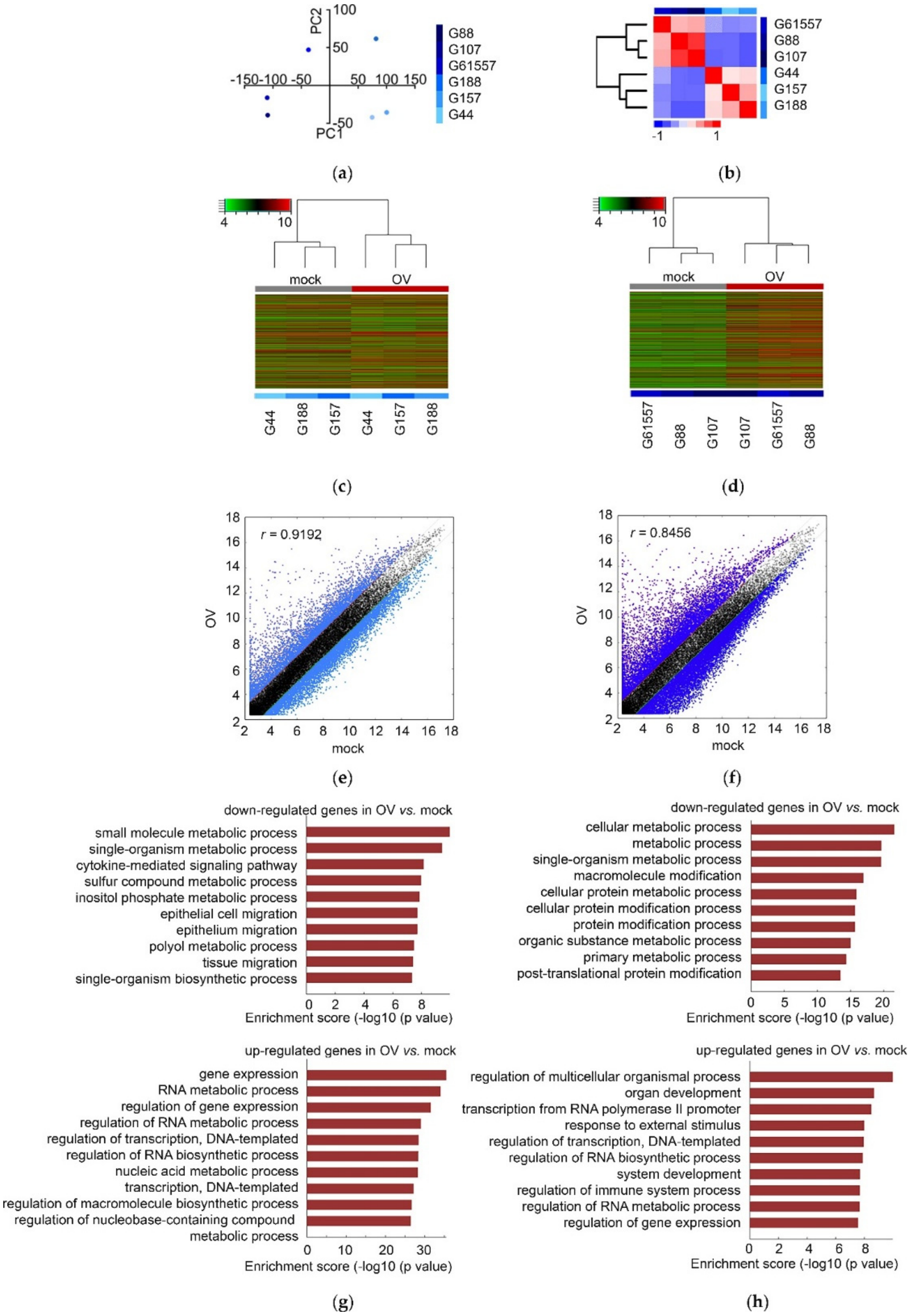

Figure S1. a,b: Principal component (PC) (**a**), and matrix correlation analysis (**b**) based on all mRNA transcripts (Arraystar™ human mRNA array, $n = 6$). **c,d:** Heatmap with unsupervised hierarchical clustering for GSCs mock or OV infected are shown separately for OV slow-responding (**c,** $n = 3$ per variant – light blue) and OV fast-responding (**d,** $n = 3$ per variant

– dark blue) GSCs based on mRNA transcripts (n = 31555 genes on Arraystar™ human mRNA array). **e,f:** Scatter plots of genes from GSCs mock, or OV infected are shown separately for OV slow-responding (**e,** n = 3 per variant, affected genes – light blue) and OV fast-responding (**f,** n = 3 per variant, affected genes – dark blue) GSCs. Pearson correlation values are disclosed. **g,h:** Gene ontology analysis of biological processes prevalent among genes deregulated upon OV infection in OV slow-responding (**g,** down-regulated – top, up-regulated – bottom), and OV fast-responding (**h,** down-regulated – top, up-regulated – bottom) GSCs.

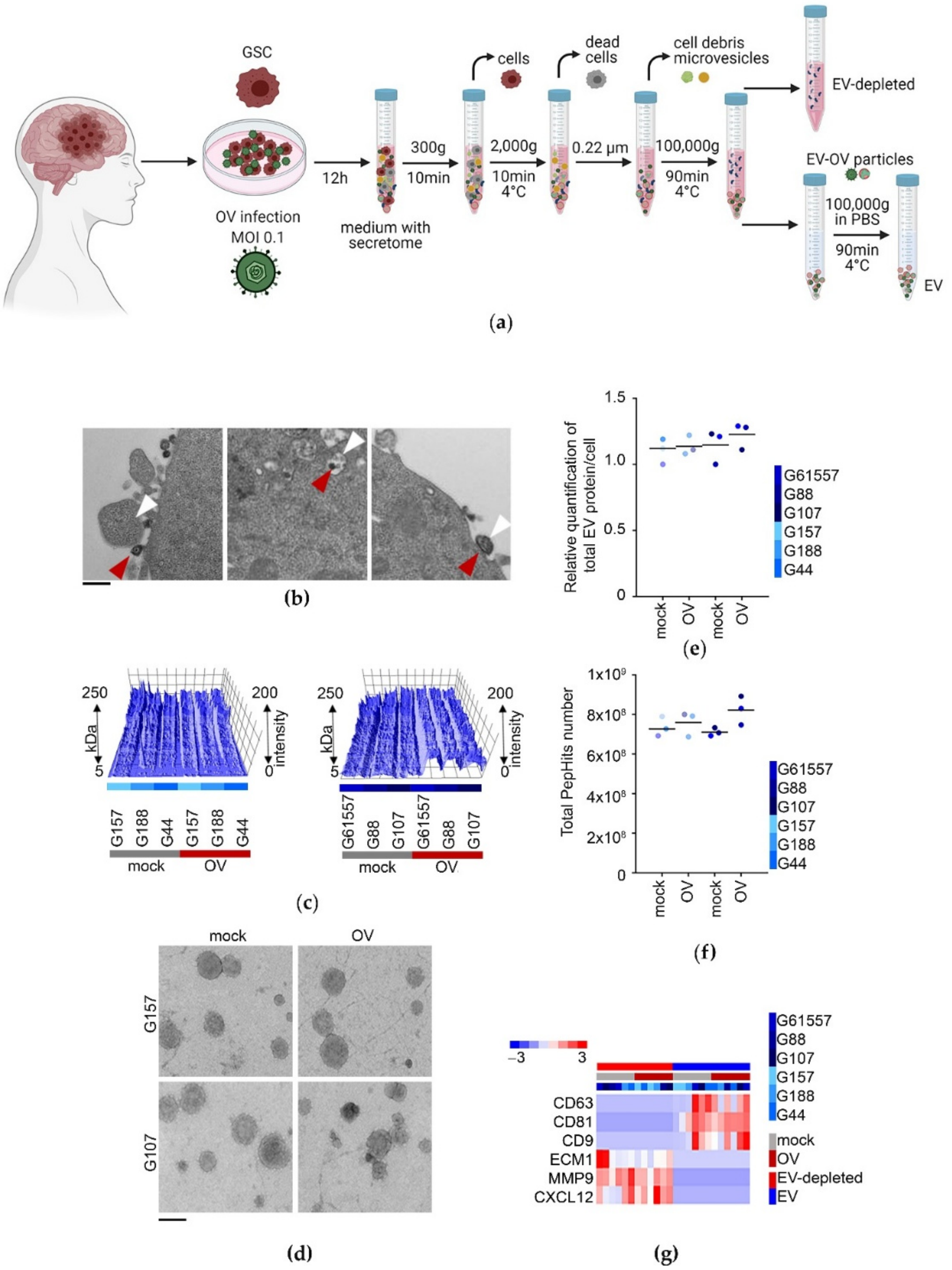

Figure S2. a: Flow chart of experimental procedures of secretome fractionation. BioRender (https://biorender.com/) software was used. **b:** Representative micrographs of OV/EV tandems by transmission electron microscopy. Red arrows - EV, white arrows – OV; scale bar: 500nm. **c:** Densitometric analysis of Coomassie Blue stained gels with EV-depleted proteins secreted by mock and OV infected GSCs. **d:** Representative micrographs of EVs secreted by mock or OV infected GSCs by transmission electron microscopy; scale bar: 500nm. **e:** Mass spectroscopy analysis of EV protein secreted by mock or OV infected GSCs by total peptide hits number. Data for human and viral proteins pooled (n = 3 per variant). **f**: Mass spectroscopy analysis of EV protein secreted by mock and OV infected GSCs by relative quantification of total protein content per cell (n = 3 per variant). **g:** Mass spectroscopy analysis of markers protein detected in EV and EV-depleted fraction respectively. Data represented by heatmap.

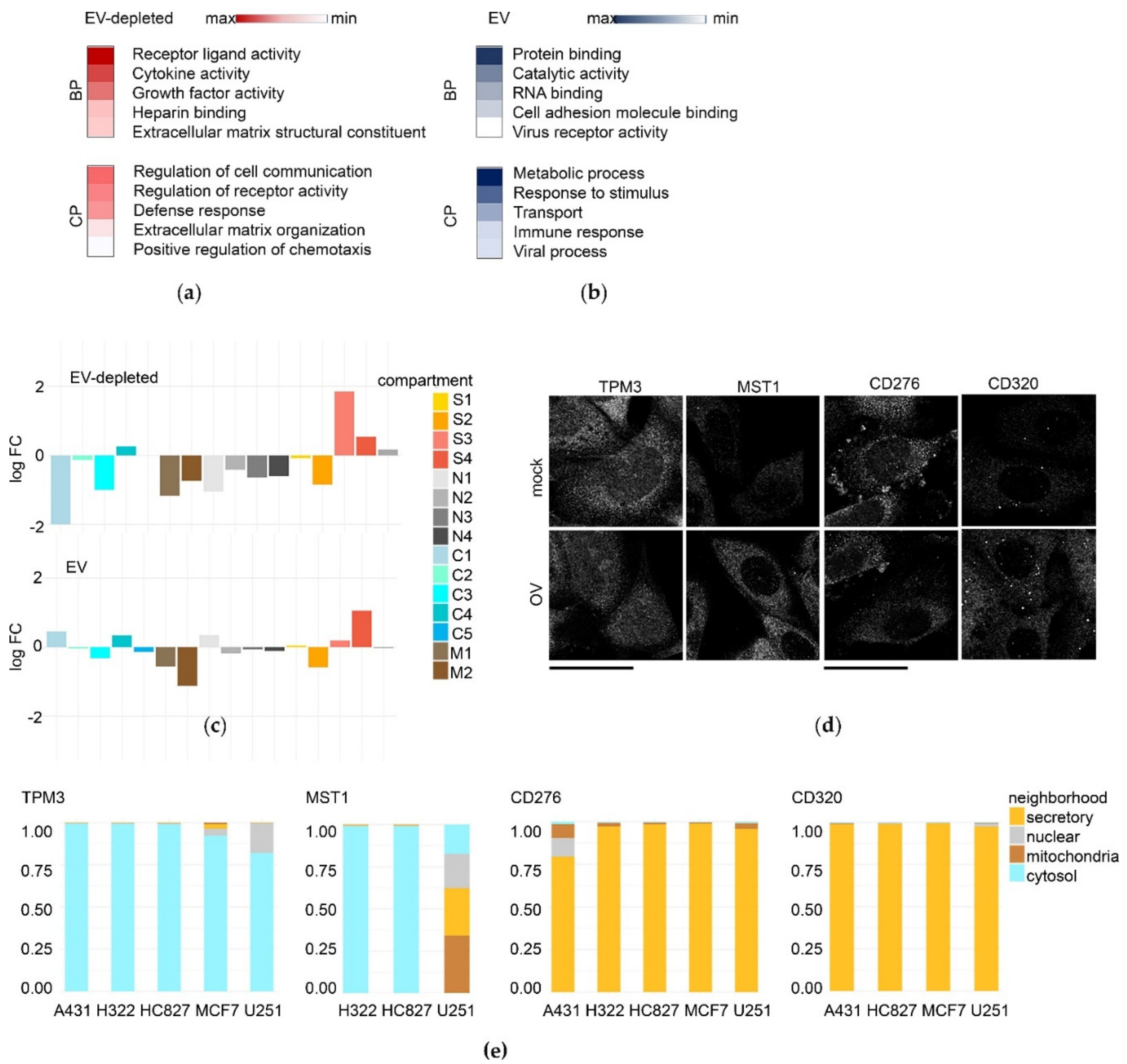

Figure S3. a,b: Gene ontology analysis of biological processes (BP) and cellular processes (CP) prevalent among proteins detected in EV-depleted (**a**), and EV (**b**) fractions of the secretome; the color bar for enrichment in each category GSCs. **c:** Bar analysis of protein localization from EV-depleted (top, $n = 1203$) and EV fractions (bottom, $n = 2088$) in Subcell Barcode dataset based on the mass-spectroscopy analysis of proteins in secretome (S), nucleus (N), cytoplasm (C), or mitochondrial (M) compartments. **d:** Representative micrographs of selected proteins in mock or OV infected GSCs by fluorescent confocal microscopy; with an antibody signal, scale bar: 25μm. **e:** Bar analysis of cell-line-specific localization of TPM3, MST1, CD276, and CD320 in Subcell Barcode dataset based on the mass-spectroscopy analysis.

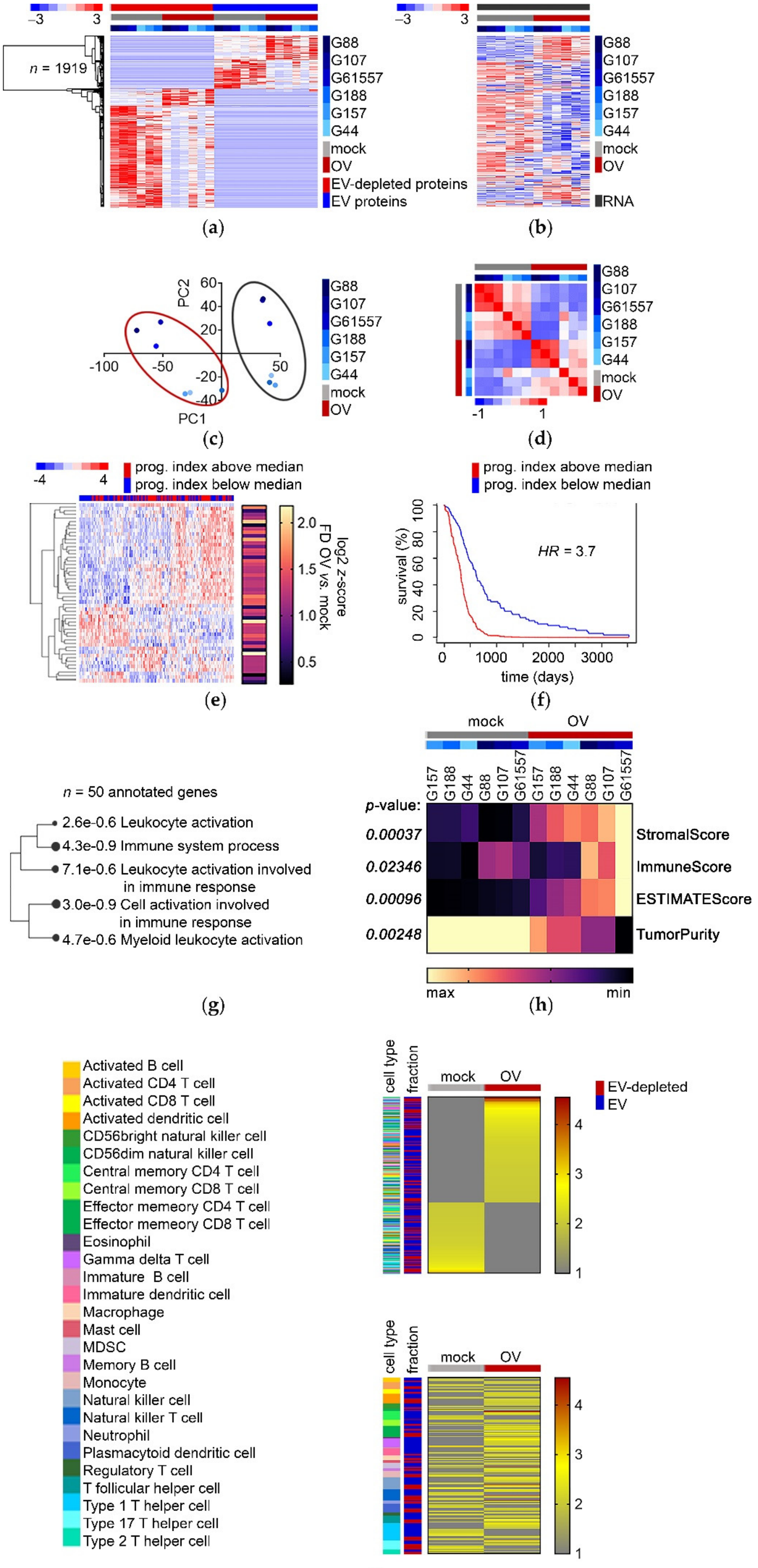

−3
3
n = 1919
G88
G107
G61557
G188
G157
G44
mock
OV
EV-depleted proteins
EV proteins
(a)
−3
3
G88
G107
G61557
G188
G157
G44
mock
OV
RNA
(b)
PC2
60
40
20
-100
-50
50
-20
-40
PC1
G88
G107
G61557
G188
G157
G44
mock
OV
(c)
G88
G107
G61557
G188
G157
G44
mock
OV
-1
1
(d)
-4
4
prog. index above median
prog. index below median
2.0
1.5
1.0
0.5
log2 z-score
FD OV vs. mock
(e)
prog. index above median
prog. index below median
survival (%)
0 20 40 60 80 100
HR = 3.7
0 1000 2000 3000
time (days)
(f)
n = 50 annotated genes
2.6e-0.6 Leukocyte activation
4.3e-0.9 Immune system process
7.1e-0.6 Leukocyte activation involved in immune response
3.0e-0.9 Cell activation involved in immune response
4.7e-0.6 Myeloid leukocyte activation
(g)
mock
OV
G157 G188 G44 G88 G107 G61557 G157 G188 G44 G88 G107 G61557
p-value:
0.00037
0.02346
0.00096
0.00248
StromalScore
ImmuneScore
ESTIMATEScore
TumorPurity
max
min
(h)
Activated B cell
Activated CD4 T cell
Activated CD8 T cell
Activated dendritic cell
CD56bright natural killer cell
CD56dim natural killer cell
Central memory CD4 T cell
Central memory CD8 T cell
Effector memeory CD4 T cell
Effector memeory CD8 T cell
Eosinophil
Gamma delta T cell
Immature B cell
Immature dendritic cell
Macrophage
Mast cell
MDSC
Memory B cell
Monocyte
Natural killer cell
Natural killer T cell
Neutrophil
Plasmacytoid dendritic cell
Regulatory T cell
T follicular helper cell
Type 1 T helper cell
Type 17 T helper cell
Type 2 T helper cell
cell type
fraction
mock
OV
EV-depleted
EV
4
3
2
1
cell type
fraction
mock
OV
4
3
2
1

(i)

Figure S4. a: Heatmap with supervised hierarchical clustering for GSCs mock or OV infected (n = 6 per variant) based on mass spectroscopy analysis in EV-depleted and EV fractions (n = 1919 proteins, cutoffs between groups mock *vs.* OV in both fractions: p-value < 0.05, FD > 2). **b:** Heatmap with unsupervised clustering for GSCs mock or OV infected (n = 6 per variant) based on mRNA transcripts (n = 1919 genes encoding for proteins shown on a panel **a**, based on Arraystar™ human mRNA array). **c,d:** Principal component (PC) (**c**), and matrix correlation analysis (**d**) based on mRNA transcripts (n = 1919 genes encoding for proteins shown on a panel **a**, based on Arraystar™ human mRNA array, n = 6 per variant). **e,f:** The list of 1500 genes (see **Fig. 4a**) was filtered out to top-50 varied genes using TCGA data associated with glioblastoma. **e**) Heatmap of genes with color annotations according to prognostic index stratification cluster (horizontal cluster) and FD upon OV infection (vertical cluster). **f**) Kaplan-Meier curves show survival analysis stratified according to their prognostic index (see panel **e**); hazard ratio (HR). **g:** A hierarchical clustering tree summarizes the biological functions of top-50 survival-predictive genes. Bigger dots indicate more significant p-values. **h:** Heatmap classify GSCs mock, or OV infected (n = 6 per variant) using four algorithms to estimate microenvironmental traits based on gene expression (n = 1500) signatures. **i:** Heatmaps classify GSCs (n = 6 per variant) to deconvolute secretome profiles into mock or OV infected cells (**top**) and immune cell type (**bottom**) of specific profiles based on immune response protein signatures (n = 220); color bars indicate the type of immune cells and EV/EV-depleted proteins.

Table S1. Information about all reagents and resources used in the preparation of the manuscript.

REAGENT or RESOURCE	SOURCE	IDENTIFIER
	Antibodies	
TPM3 Rabbit RRID AB_2633251	Thermo Fisher	720306
MST1 Rabbit RRID AB_11157025	Thermo Fisher	PA5-22015
CD276 Rabbit RRID AB_2789731	Thermo Fisher	PA5-82573
CD320 Rabbit RRID AB_2664869	Thermo Fisher	PA5-67296
CD63 Mouse (TS63)	Abcam	AB59479
Secondary Goat anti-Rabbit polyclonal	Thermo Fisher	32460
	Bacterial and Virus Strains	
BL21 Star (DE3) Chemically Competent *E. coli*	Thermo Fisher	C601003
Oncolytic herpes simplex virus type 1 (oHSV) rQNestin34.5	48	NA
	Biological Samples	
Patient-derived GSCs	49	N/A
	Chemicals, Peptides, and Recombinant Proteins	
EGF	Pepro Tech	AF-100-15
FGF2	Pepro Tech	100-18C
B27	Gibco	21103049
Neurobasal	Gibco	21103049
TRIzol™ Reagent	ThermoFisher	15596018
	Critical Commercial Assays	
Power Up SYBR Green Master Mix	Thermo Fisher	A25743
TaqMan Fast Advanced Master Mix	Thermo Fisher	4444963
Agilent whole human genome microarray	Arraystar	Agilent-026652 4x44K v2
iScript™ cDNA Synthesis Kit	Bio-Rad	1708890
	Deposited Data	
Mass spectrometry	This paper	Supplemental spreadsheets Table 2
Genome microarray	This paper	Deposition: GSE155247
	qPCR primers:	

TPM3:<br>F: 5'-GA-<br>GAGGTATGAAGGTTATTCA-3'<br>R: 5'-ATCACCACCTTAC-<br>GAGCCACC-3'<br>MST1:<br>F: 5'-GACAGCCCTCACGTAGTCAA -<br>3'<br>R: 5'-AGGAGCCATCCAAAACGGG-3'<br>CD276:<br>F: 5′-<br>GTGGGGCTGTCTGTCTGTCTCAT-3′<br>R: 5′-CTGTCAGAG-<br>TGTTTCAGAGGCT-3′<br>CD320:<br>F: 5′-CTGCGACAGGGACTTGGA-3′<br>R: 5′-GGGTACATGGCTCAATCCTG-<br>3′<br>18S rRNA:<br>F: 5′-GGCCCTGTAATT-<br>GGAATGAGTC-3′<br>R: 5′-CCAAGATCCAACTAC-<br>GAGCTT-3′	Invitrogen	N/A
Recombinant DNA		
pCSCMV PalmtdTomato	50	N/A
Software and Algorithms		
dchip with R package version dChip 2010.01	51	https://sites.google.com/site/dchip-soft/home
ShinyGO v0.61	52	http://bioinformatics.sdstate.edu/go/ accessed on: 01.03.2019
GBM biodiscovery portal	53	https://gbm-biodp.nci.nih.gov accessed on: 01.03.2019
GlioVis:	54	http://gliovis.bioinfo. accessed on: 15.01.2020
TCGA GBM	28	https://www.cancer.gov/ accessed on: 01.03.2020
Subcellbarcode	55	http://www.subcellbarcode.org
BioRender	NA	https://biorender.com/

Table S2. Mass spectroscopy analysis of two protein fractions EV-depleted and EV: please see the link.
